# Anxiety and obsessive-compulsive disorder (OCD) in adults with CHARGE syndrome

**DOI:** 10.1186/s11689-026-09673-5

**Published:** 2026-03-02

**Authors:** Shanti A. Madhavan-Brown, Timothy S. Hartshorne, Sarah B. Schneider, Lillian J. Slavin

**Affiliations:** 1 Department of Psychological Science, Albion College, Albion, MI USA; 2 Department of Psychology, Central Michigan University, Mount Pleasant, MI USA; 3 Division of Behavioral Medicine and Clinical Psychology, Cincinnati Children’s Hospital Medical Center, Cincinnati, OH USA; 4 Nemours Children’s Hospital, Delaware, Wilmington, DE USA

**Keywords:** CHARGE syndrome, Anxiety, Obsessive-compulsive disorder, Pain, Sleep, Behavioral phenotype

## Abstract

**Background:**

CHARGE syndrome is a rare genetic disorder with multiple physical, cognitive, behavioral, and sensory impairments. Anxiety is a common finding. Difficulties with pain, sleep, sensory impairment, communication, daily stress, and unpredictable environments are potential contributing factors to this anxiety. Further research is needed to gain a better understanding of the presentation of anxiety in CHARGE syndrome to promote proper diagnosis and treatment of anxiety.

**Methods:**

An on-line survey was distributed to adults and guardians of adults with CHARGE syndrome. Fifty-two participants provided responses to the *Developmental Behavior Checklist-Parent Version*, the *Florida Obsessive-Compulsive Inventory*, and the *Generalized Anxiety Disorder 7-Item Scale*. Participants also provided demographic data, diagnostic characteristics of CHARGE, their perception of their anxiety, diagnosed mental health disorders, and the frequency of other potential factors of anxiety (i.e. pain and sleep concerns). Descriptive statistics, independent samples t-tests, and Pearson’s correlations provided information on the presentation of anxiety and the relationships between potential factors and anxiety subscale scores. A hierarchical multiple linear regression analysis and mediation analysis were used to investigate if sleep mediates the relationship between pain and anxiety.

**Results:**

Generalized anxiety disorder and obsessive-compulsive disorder were the first (39% of sample) and fourth (27% of sample) most common mental health diagnoses reported and 50% of the sample had been diagnosed with at least one anxiety disorder. The most commonly reported anxious behaviors included getting obsessed with an idea or activity, being impatient, getting upset or distressed over small changes in the routine or environment, and being tense, anxious, or worried. Sleep was found to mediate the relationship between pain and anxiety.

**Conclusions:**

This study has implications for understanding the behavioral phenotype of CHARGE syndrome. Based on self-report and legal guardian report, anxiety is a common experience among individuals with CHARGE; generalized anxiety disorder and obsessive-compulsive disorder diagnoses/behavior are the most frequently reported. Further research into the management of pain and improvement of sleep as anxiety interventions for individuals with CHARGE may prove fruitful.

## Background

CHARGE syndrome is a rare (approximately 1 in every 10,000 live births) congenital disorder with multiple physical, cognitive, behavioral, and sensory impairments. The majority of cases are caused by a *CHD7* gene mutation [1] and it is the leading cause of congenital deafblindness. The presence of major and minor anomalies is frequently used as an accurate method of clinical diagnosis [2]. The major criteria are described as the four “C’s” of CHARGE (coloboma, choanal atresia, cranial nerve anomalies, characteristic CHARGE inner & outer ear) with many other minor criteria used in diagnosis [3, 4]. Cognitive functioning and adaptive behavior levels are highly variable across the population and difficult to assess due to multiple confounds (e.g., hearing impairment, vision impairment, fatigue, medical conditions, etc.; [5]). It is possible that assessment of the cognitive abilities of adults with CHARGE underestimates cognitive functioning due to the symptoms of CHARGE greatly impacting the communication skills of some individuals [5]. Several research studies have also measured the prevalence of neurodevelopmental disorders among individuals with CHARGE [6]. Prevalence rates of attention-deficit/hyperactivity disorder (ADHD; 11%−26%; [6–8]) as well as autism spectrum disorder (ASD; 14%−30%; [6, 8, 9]) have been reported across several studies. The prevalence of psychiatric diagnoses given to individuals with CHARGE has also been studied.

### Anxiety and obsessive-compulsive disorder within CHARGE syndrome

Previous research, while limited, indicates obsessive-compulsive disorder (OCD) and anxiety disorders are among the top three most common mental health diagnoses among individuals with CHARGE syndrome. Blake et al. [7] found that 43% of caregivers of individuals with CHARGE reported that their child had an OCD diagnosis and 37% indicated an anxiety disorder diagnosis. Wachtel et al. [8] found that 19.5% of participants endorsed an anxiety disorder diagnosis and 17.2% endorsed an OCD diagnosis. N. Hartshorne et al. [9] found that 47% of adolescents and adults with CHARGE endorsed obsessive-compulsive behaviors and 45% endorsed anxiety. In a survey of parents and guardians of children and adolescents with CHARGE syndrome, Madhavan-Brown [10] found that OCD (11%) and generalized anxiety disorder (GAD; 7.5%) were the third and fourth most common mental health diagnoses reported and 16% of the total sample had been diagnosed with at least one anxiety disorder. Taken together, this research suggests that anxiety and related diagnoses are prevalent concerns in those with CHARGE syndrome and, therefore, can have a significant impact on the behavior of these individuals [11].

Many of the most common anxious behaviors [12, 13] are also exhibited by individuals with CHARGE syndrome, particularly self-injurious behavior, sleep problems, and repetitive behaviors [7, 10, 11, 14, 15]. Hartshorne and Cypher [15] found that 36% of their 100 participants engaged in repetitive or compulsive behavior characteristic of OCD, despite only three participants having an official, clinical diagnosis. In another study, parents of children with CHARGE reported “obsession with an idea or activity” as occurring the most often [10]. Bernstein & Denno [14] found that their participants with CHARGE received an above average score on the *Compulsive Behavior Checklist* [16] and 72% of participants engaged in repetitive behavior for an hour or more each day. A majority of the participants reported that these behaviors negatively interfered with social activities, relationships, and daily routines.

Repetitive behavior is recognized as part of the CHARGE syndrome behavioral phenotype [17]. Repetitive behavior among individuals with CHARGE is hypothesized to be correlated with anxiety, as it has been observed to increase during times of stress [11]. Adults with CHARGE show higher rates of OCD symptomatology and diagnosis (i.e., 11–43% prevalence; [6, 7, 10]) compared to the general population (i.e., 2.3% incidence; [18]). Preliminary research has also found a similar pattern in children with CHARGE [10]. Thus, obsessions and (particularly) compulsive behaviors are commonly reported among individuals with CHARGE and updated research on the prevalence of OCD diagnoses in individuals with CHARGE would be beneficial.

### Etiology of anxiety and OCD within CHARGE syndrome

It has been suggested that the common physical, cognitive, and behavioral characteristics associated with CHARGE syndrome can lead to higher levels of anxious behavior [8, 11]. Some of these characteristics include pain, sleep concerns, and sensory impairment. Difficulties with communication, stress, and lack of predictability in the environment could also be contributing factors.

Individuals with developmental disabilities experience a higher number of health conditions (e.g., constipation, gastroesophageal reflux disease, sleep problems) compared to the overall population [15, 17, 19, 20]. Furthermore, people with CHARGE often contend with a high number of hospitalizations and surgeries [15, 21]. Stratton and Hartshorne [21] found parents of children with CHARGE reported that a large number experienced pain at high frequencies. Research suggests that increased prevalence of challenging behaviors and anxiety among individuals with developmental disabilities (and/or CHARGE) could be a result of their overall health, medical conditions, and level of pain [19, 20]. Pain has been described as an important—if not the most important—influence on the behavior of individuals with CHARGE [11].

Over half of the CHARGE population has reported sleep problems (i.e., 59% incidence; [8]) including: obstructive sleep apnea, initiation or maintenance of sleep, and difficulties sleeping due to visual impairment [22]. Research has shown that lack of sleep is linked to increases in challenging behaviors [19, 23]. The relationship between anxiety and sleep has been seen as reciprocal; a pattern seen in survey research of adolescents and adults with CHARGE [9]. Given the high incidence of sleep problems among individuals with CHARGE syndrome and the apparent association between sleep problems and anxiety, it is not surprising that there is a common concern of anxiety among those with CHARGE.

Individuals with CHARGE syndrome can experience deficits in some or all of the seven sensory systems [24] which, in turn, have a significant impact on behavior [8, 11]. The environment is often unpredictable—and potentially anxiety-provoking—to individuals with CHARGE because they have a reduced ability to efficiently or effectively gather information about the environment from their sensory systems. Challenges with communication are also common in the CHARGE population [4]. Difficulties with communication, coupled with multi-sensory impairment, make it difficult for these individuals to explore, obtain, comprehend, and use information from their environment [25]. This lack of predictability and daily stress can result in higher levels of anxiety [11, 26–28]. Thus, individuals with CHARGE experience many of the common causes of anxiety.

It has been suggested that the underlying factors that contribute to anxiety also contribute to the presentation of OCD behaviors in CHARGE as well. In other words, higher levels of pain, sleep concerns, sensory impairment, and communication delays increase the stress and uncertainty in the environment which leads to higher levels of obsessions and compulsive behaviors to combat this anxiety [8, 10, 11]. Those with CHARGE syndrome might also be at more risk for behavioral and psychiatric concerns more broadly due to the genetic cause of the syndrome, increasing the risk of anxiety diagnoses and OCD [8]. Wachtel, Hartshorne, and Dailor [8] also described how the multiple sensory impairments characteristic of CHARGE can negatively impact interpersonal interactions leading to the use of maladaptive behaviors (e.g., obsessions, compulsions, repetitive behaviors, self-injurious behaviors) to communicate or contend with stress in their environment.

### Research questions

Research on anxiety and anxious behavior among individuals with CHARGE syndrome is limited. Studies have indicated that particular subtypes of anxiety can vary between genetic syndromes or developmental disorders [29–32] but CHARGE has yet to be included. Preliminary research suggests that CHARGE syndrome might have uniquely higher levels of OCD-related behavior than individuals with other genetic syndromes and typically developing children with an anxiety diagnosis [10]. The etiology of anxiety may be more nuanced in low-incidence populations [30], therefore, further analysis of the types and frequency of anxious behavior exhibited by persons with CHARGE syndrome is needed to develop a better understanding of how anxiety manifests among adults with CHARGE. This analysis could help inform diagnosticians when making a mental health diagnosis for an individual with CHARGE.

There were three research questions addressed in this study:

- What is the prevalence of anxiety and obsessive-compulsive diagnoses among adults with CHARGE Syndrome?
- What is the presentation of anxious and obsessive-compulsive disorders in individuals with CHARGE syndrome (including the types and frequency of anxious behavior)?
- How do pain and sleep contribute to anxiety in individuals with CHARGE?

## Methods

### Participants

Adults with CHARGE syndrome (*ADULT* group; *n* = 32, *M*_age_ = 29.03, 68% female) and legal guardians of adults with CHARGE syndrome (*LG* group; *n* = 20, *M*_age_ = 27.5, 55% female) were recruited and created a combined participant group (*COMBINED* group). Five participant responses were removed because they did not meet the inclusion criteria (i.e., no diagnosis of CHARGE syndrome, completed the incorrect survey, did not meet age criteria) leaving the final *COMBINED* participant group (*n* = 52, *M*_age_ = 28.47, 63% female). All adults in the *COMBINED* group reported at least one major or minor characteristic of CHARGE (with many indicating several). The characteristics of CHARGE reported the most included: hearing impairment, visual impairment, developmental delay, and cranial nerve abnormalities or dysfunction. See Table 1 for demographic information.

**Table 1:** Demographic information

		ADULT group (*n* = 32)	LG group (*n* = 20)	COMBINED group (*n* = 52)
Tested positive for CHD7 gene mutation	*n* (%)	14 (44%)	9 (45%)	23 (44%)
Tested negative for CHD7 gene mutation	*n* (%)	3 (9%)	3 (15%)	6 (12%)
Hearing impairment	*n* (%)	29 (91%)	19 (95%)	48 (92%)
Visual impairment	*n* (%)	26 (81%)	18 (90%)	44 (85%)
Coloboma of the eye	*n* (%)	23 (72%)	17 (85%)	40 (77%)
Heart defects or malformations	*n* (%)	18 (56%)	16 (80%)	34 (65%)
Choanal atresia or stenosis	*n* (%)	17 (53%)	10 (50%)	27 (52%)
Growth delay	*n* (%)	24 (75%)	13 (65%)	37 (71%)
Genital hypoplasia	*n* (%)	19 (59%)	14 (70%)	33 (64%)
Ear abnormalities	*n* (%)	23 (72%)	18 (90%)	41 (79%)

A majority of respondents in the *ADULT* group completed the survey by themselves (84.4%, *n* = 27) with a much smaller group completing the survey with the help of a parent, guardian, or interpreter (15.6%, *n* = 5). Participants were asked to describe the ways that they communicate with others. Almost all of the *ADULT* group indicated that they used oral speech (96.8%, *n* = 31) whereas a smaller amount indicated use of sign-language (25%, *n* = 8), text-based speech (34.4%, *n* = 11), or some combination of the three. All of the respondents in the *LG* group were legal guardians of adults with CHARGE who will filled out the survey. When providing information about communication skills, only one participant indicated that their adult with CHARGE “makes reactions or noises or behaviors which can be difficult to interpret” (3%) while more indicated that their adult with CHARGE “uses behaviors such as gestures, sounds, body movements” (18.8%, *n* = 6), “uses single words, signs, picture symbols, or object symbols to represent basic needs” (12.5%, *n* = 4), “uses some 2- to 5-word phrases and sentences using speech, signs, picture symbols, etc.” (9.3%, *n* = 3), or “uses verbal or sign language in complete sentences” (18.8%, *n* = 6).

### Measures

Participant responses were collected during the beginning of the COVID-19 pandemic (Spring/Summer 2020). Two questions were asked to gather participant’s perceptions on how this impacted their levels of anxiety. All participants (the *COMBINED* group) completed the CHARGE Syndrome Demographic/Anxiety Questionnaire and the Developmental Behavior Checklist-Parent Version (DBC-P; [33]). The *ADULT* group also completed the Florida Obsessive-Compulsive Inventory (FOCI) and the Generalized Anxiety Disorder 7-Item Scale (GAD-7; [34]).

Most of the CHARGE Syndrome Demographic/Anxiety Questionnaire items were adapted from previous research studies of individuals with CHARGE [35] and gathered basic demographic data and information about the diagnostic characteristics of CHARGE [2, 36]. Additional questions were included that asked about individual’s ability to hear, ability to see, age of walking, number of surgeries, and communication skills. Participants were asked “How often do you [think your adult] experience[s] pain (e.g., migraines, abdominal migraines, surgery pain, constipation, ear infections, etc.)?” and asked to provide an estimate of frequency on a 7-point Likert scale (with answers ranging from “Once every few months” to “Once a day”). A similar question asked about the frequency of difficulties with sleep: “How often do you [think your adults] have[has] difficulties with sleep (e.g., trouble falling asleep, trouble staying asleep, waking up multiple times in the night, etc.)?”. Questions regarding anxiety were designed for this study. Additionally, information about whether the adult had been diagnosed with a mental health disorder and/or anxiety disorder was collected. These questions allowed us to gather an understanding of other anxious behaviors that adults with CHARGE may be exhibiting that are not captured in the anxiety scales used.

The DBC-P [33] is a rating scale used to assess the frequency of behavioral and emotional concerns of individuals with developmental and cognitive disabilities aged 4–18 years. As the focus on this study was anxiety, only the items from the DBC-P Anxiety subscale (9 items; Cronbach’s alpha for current study: α = 0.66), DBC-P Anxious Behavior Rating Scale (ABRS; 7 items; Cronbach’s alpha for current study: α = 0.69) and additional items that mapped onto an anxiety diagnosis (28 items total, total possible raw score = 84; Cronbach’s alpha for current study: α = 0.86) were selected for the questionnaire. All participants (*COMBINED* group) completed the DBC-P, but the items were edited to use fist-person language for the *Adult* group survey. The DBC-P has also been used to research the behaviors of individuals with genetic syndromes [29].

The FOCI [37] is a self-report questionnaire used to assess the presence and severity of symptoms related to obsessive-compulsive disorder (OCD). This is accomplished by two concurrent parts of the scale: a symptom checklist (The Checklist; K-R 20 = 0.83; Current study K-R 20 is: α = 0.86) and a Severity Scale (SS; α = 0.89; Cronbach’s alpha for current study: α = 0.88). This scale was selected as a behavior checklist for adults with CHARGE to provide information on their own OCD related behaviors. Prior research has indicated that the FOCI is highly correlated with the Yale-Brown Obsessive Compulsive Scale Self-Report (Y-BOCS-SR; [38]) and has good internal consistency - with the overall scale and both components [37, 39, 40].

The GAD-7 [34] is a self-report rating scale intended to assess symptomology of Generalized Anxiety Disorder. This scale was selected because it is a brief measure and would give an indication of the frequency of GAD symptoms in adults with CHARGE syndrome. The seven items of the GAD-7 (α = 0.92; Cronbach’s alpha for current study: α = 0.93) are rated on a 4-point Likert scale based on the frequency in the last two weeks. The GAD-7 has a sensitivity of 89% and 82% specificity for GAD [34]. A meta-analysis of 11 studies [41] indicated that the scale was effective at identifying GAD using a cut score between 7 and 10 (with the optimal sensitivity and specificity at a cut score of 8). Additional studies have indicated that it has convergent validity with the Beck Anxiety Inventory and the anxiety subtest of the Symptom Checklist-90 [41–44].

### Procedure and ethics

An invitation to participate was distributed through Facebook pages specific to CHARGE syndrome and by the CHARGE Syndrome Foundation. Qualtrics^®^ was used to develop, distribute, and collect the survey results. The responses of adults with CHARGE and parents/guardians were analyzed separately and there was no attempt to link parent and adult child responses. Indeed, measures were put in place to have the survey answered only once per family unit. The procedures, informed consent form, and all materials from this project were submitted and approved by the Institutional Review Board (IRB) of Central Michigan University in accordance with the Declaration of Helsinki. Informed consent to participate in the study was received from each participant. Clinical trial number: not applicable.

### Data analysis

To analyze the prevalence of anxiety and obsessive-compulsive disorders, descriptive statistics on the reported mental health diagnoses, GAD-7 results, and FOCI results were analyzed and compared to the prevalence rates among the general population provided by the National Institute of Mental Health. To determine the manifestation of anxiety and obsessive-compulsive disorder symptoms, the frequency of responses to each item on the DBC-P, GAD-7, FOCI, and perceived anxiety questions was also gathered. Lastly, in order to answer the third research question (impact of pain and sleep on anxiety), independent samples t-tests and Pearson’s correlations were calculated to determine the relationships between the potential factors, anxiety subscale scores, and the scores of both participant groups. A hierarchical multiple linear regression analysis and a mediation analysis were used to investigate if sleep mediates the relationship between pain and anxiety. The indirect effect was tested using a percentile bootstrap estimation approach implemented with the PROCESS macro Version 3 [45]. This model estimated the direct effects of pain on anxiety and indirect change in anxiety once sleep was added to the model. The indirect effect of pain on anxiety after mediation by sleep was considered significant if the 95% confidence interval (CI) of the estimated effect does not include zero.

## Results

### Prevalence of anxiety disorders

The most common mental health disorders for the *COMBINED* group were generalized anxiety disorder (GAD), major depressive disorder (MDD), attention-deficit/hyperactivity disorder (ADHD), and obsessive-compulsive disorder (OCD). Autism spectrum disorder (ASD) was also a more common diagnosis in the *LG* group. From the *COMBINED* group, 26 participants (50%) noted at least one anxiety disorder and/or obsessive-compulsive disorder diagnosis; 13 participants (25%) noted more than one anxiety disorder diagnosis. Table 2 displays the results for the *ADULT*, *LG*, and *COMBINED* groups.

**Table 2:** Mental health disorder diagnoses

Mental Health Disorder Diagnosis	ADULT group *n* (%)	LG group *n* (%)	COMBINED group *n* (%)
Generalized Anxiety Disorder	14 (44%)	6 (30%)	20 (39%)
Major Depressive Disorder	14 (44%)	3 (15%)	17 (33%)
Attention-Deficit/Hyperactivity Disorder	7 (22%)	8 (40%)	15 (29%)
Obsessive-Compulsive Disorder	7 (22%)	7 (35%)	14 (27%)
Post-Traumatic Stress Disorder	4 (13%)	4 (20%)	8 (15%)
Autism Spectrum Disorder	0 (0%)	7 (35%)	7 (14%)
Social Anxiety Disorder	5 (16%)	0 (0%)	5 (10%)

The *ADULT* group provided responses on assessment scales for GAD (GAD-7) and OCD (FOCI). 50% of participants (*n* = 16) indicated on the GAD-7 that their symptoms made it somewhat difficult for them to function in their home life, work settings, and/or relationships. A quarter of the respondents indicated no difficulty resulting from their symptoms (*n* = 7, 25%). Another quarter indicated that their symptoms made it very or extremely difficult (*n* = 7, 25%). Based on the responses from the *ADULT* group, 63% (*n* = 20) of participants met the criteria for further evaluation of GAD. The FOCI symptom checklist provides a total score of obsession and compulsion traits (ranging from 0 to 20) with a score of eight or more indicating the potential for symptomology of OCD. Eleven participants (34%) indicating eight or more obsessions and/or compulsions. On the Severity Scale of the FOCI, 28% of participants (*n* = 9) indicated that their symptoms had moderate to extreme impact on their level of distress, cognitions, and daily living activities.

### Anxious behavior types and frequencies

After being given a definition of anxiety, respondents in the *LG* group were asked if they thought that their adult with CHARGE syndrome experiences anxiety while participants in the *ADULT* group were asked if they thought they had anxiety. 90% of participants (*n* = 47) in the *COMBINED* group answered yes. These participants rated how frequently this anxiety was expressed and 68% (*n* = 32) indicated that this anxiety was experienced several times a week or more frequently (Table 3). The participants in the *COMBINED* group who indicated that they experience anxiety (*n* = 47) were also asked to indicate how this level of anxiety was impacted by the COVID-19 pandemic. The respondents reported that the anxiety increased (*n* = 28, 60%), stayed the same (*n* = 16, 34%), or decreased (*n* = 3, 6%), since the start of the pandemic.

**Table 3:** Reported frequency of anxiety

Frequency of Anxiety	ADULT group *n* (%)	LG group *n* (%)	COMBINED group *n* (%)
At least once per day	10 (31%)	6 (30%)	16 (30%)
Several times a week	9 (28%)	7 (35%)	16 (30%)
Once a week	3 (9%)	3 (15%)	6 (12%)
A couple times a month /Every other week	5 (16%)	1 (5%)	6 (12%)
Once a month	1 (3%)	1 (5%)	2 (4%)
Once every few months	0 (0%)	1 (5%)	1 (2%)
No anxiety	4 (13%)	1 (5%)	5 (10%)

All 52 participants completed selected items from the DBC-P, which were chosen because they described behaviors related to anxiety and internalizing behavior and included a 7-item anxiety behavior rating scale [ABRS] from the DBC-P. The behaviors that the majority of participants indicated as the most *frequently* reported (i.e., very/often true) included getting obsessed with an idea or activity, being impatient, and getting upset or distressed over small changes in the routine or environment, and being tense, anxious, or worried. Three DBC-P items, that match criteria for OCD from the DSM-5 (Diagnostic and Statistical Manual of Mental Disorders, 5th Edition) were rated by participants as behavior that their child with CHARGE syndrome exhibits sometimes or very often (i.e., gets obsessed with an idea or activity [92%], preoccupied with only one or two particular interests [77%], arranging objects or routine in a strict order [83%]). Indeed, obsession with particular ideas or activities was rated the most frequent out of all of the behaviors, with 54% of participants indicating that this behavior was very frequently shown.

The *ADULT* group responses to the GAD-7 and FOCI also provided information on the types and frequency of anxious behavior reported by adults with CHARGE syndrome. The behavior indicated as the most frequently occurring (i.e., Nearly every day) on the GAD-7 was “worrying too much about different things”. On the first part of the FOCI (i.e., a checklist of common obsessions and compulsions), “worry about harm coming to a love one” and “worry about losing something valuable” were the most *frequently* reported obsessions while “feeling a need to ‘confess’” or “repeatedly asking for reassurance that you said or did something correctly” was the most *frequently* reported compulsion.

### Pain and sleep impacts on anxiety of adults with CHARGE syndrome

Respondents in the *ADULT* group were asked to report the frequency of their pain (e.g., migraines, abdominal migraine, surgery pain, constipation, ear infections, etc.) and difficulties with sleep (e.g., trouble falling asleep, trouble staying asleep, waking up multiple times in the night, etc.). The *LG* group was asked to provide the same information regarding their adult with CHARGE syndrome. There was a wide range of reported frequency of pain and sleep concerns (see Table 4) that ranged from once every few months to at least once a day. Participants were also surveyed on their perceived level of anxiety (see Table 3), the DBC-P Anxiety Behavior Rating Scale (ABRS) and an DBC-P anxiety subscale. Independent samples *t*-tests were conducted to analyze the differences in means between the two participant groups surveyed and results indicated no significant differences between the means. Therefore, the two participant groups were combined (into the *COMBINED* group) for further analysis of the relationship between these variables.

**Table 4:** Reported frequency of pain and sleep concerns

Frequency	COMBINED group Pain Concerns *n* (%)	COMBINED group Sleep Concerns *n* (%)
At least once per day	8 (16%)	14 (27%)
Several times a week	8 (16%)	12 (24%)
Once a week	4 (8%)	5 (10%)
A couple times a month /Every other week	9 (17%)	6 (12%)
Once a month	8 (16%)	4 (8%)
Once every few months	14 (27%)	9 (18%)

Correlations were conducted to determine the relationships between the potential contributing factors of anxious behavior (i.e., pain and sleep; see Table 5). Higher scores for each scale indicated higher level (i.e., higher frequency) of concerns. The results indicated no significant relationship between a few of the scales (e.g., pain and the Anxiety Subscale score, and pain and the perception of anxiety frequency). In contrast, a significant correlation between pain and sleep was found, indicating that higher pain was associated with increased sleep difficulties (*r*[48] = 0.45, *p* <.001). Positive correlations were found between the ABRS and the Anxiety Subscale (*r*[50] = 0.91, *p* <.001), the ABRS and anxiety frequency perception (*r*[50] = 0.54, *p* <.001), and the Anxiety Subscale and anxiety frequency perception (*r*[50] = 0.45, *p* <.001). Participants who reported higher frequency of anxious behavior on one measure also reported higher levels of anxiety on the other measures in the survey.

**Table 5:** Correlations

Scale	1	2	3	4	5
1. Pain	—				
2. Sleep	0.45**	—			
3. ABRS	0.33*	0.39**	—		
4. Anxiety Subscale	0.22	0.34*	0.91**	—	
5. Anxiety Perception	0.28	0.30*	0.54**	0.45**	—

Pain = reported pain difficulties, Sleep = reported sleep difficulties, ABRS = DBC-P Anxiety Behavior Rating Scale, Anxiety Subscale = DBC-P Anxiety Subscale, Anxiety Perception = reported perceived anxiety

* indicates significance at the 0.05 level

** indicates significance at the 0.01 level

A positive correlation between pain and the ABRS (*p* =.01) suggested that higher levels of pain was associated with higher anxiety. Similarly, a significant correlation between sleep and each measure of anxiety was found (with the ABRS, *p* =.005; with the Anxiety Subscale, *p* =.013; and with participant perception of anxiety frequency, *p* =.03). This indicated that higher sleep difficulties were associated with higher anxiety on each measure of anxious behavior. These results suggest that higher levels of pain and sleep concerns are significantly related to higher levels of anxiety.

To further analyze these relationships, a two-step hierarchical multiple linear regression analysis was conducted to assess predicting factors of anxiety (measured by the ABRS). Pain was entered into the regression equation as the variable for Step 1 of this analysis to control for the participants’ reported pain frequency (see Table 6). The results indicated that pain accounted for 11.9% of variance in the ABRS score and the regression model was significant, *F*(1,44) = 6.50, *p* =.014. At Step 2, we entered the sleep difficulty variable and it predicted an additional 7.1% of variance and also created a significant model, *F*(2,45) = 5.51, *p* =.007.

**Table 6:** Hierarchical multiple linear regression analysis predicting anxiety (ABRS)

Step and Predictor Variable	*b(β)*	*SE*	*ΔR* ^*2*^
Step 1			0.11*
Pain	0.56 (0.34)*	0.22	
Step 2			0.07*
Pain	0.34 (0.20)	0.24	
Sleep	0.48 (0.29)*	0.24	

Pain = reported pain difficulties, Sleep = reported sleep difficulties, ABRS = DBC-P Anxiety Behavior Rating Scale. Higher scores indicated higher levels of concern

* indicates significance at the 0.05 level

**Fig. 1:**
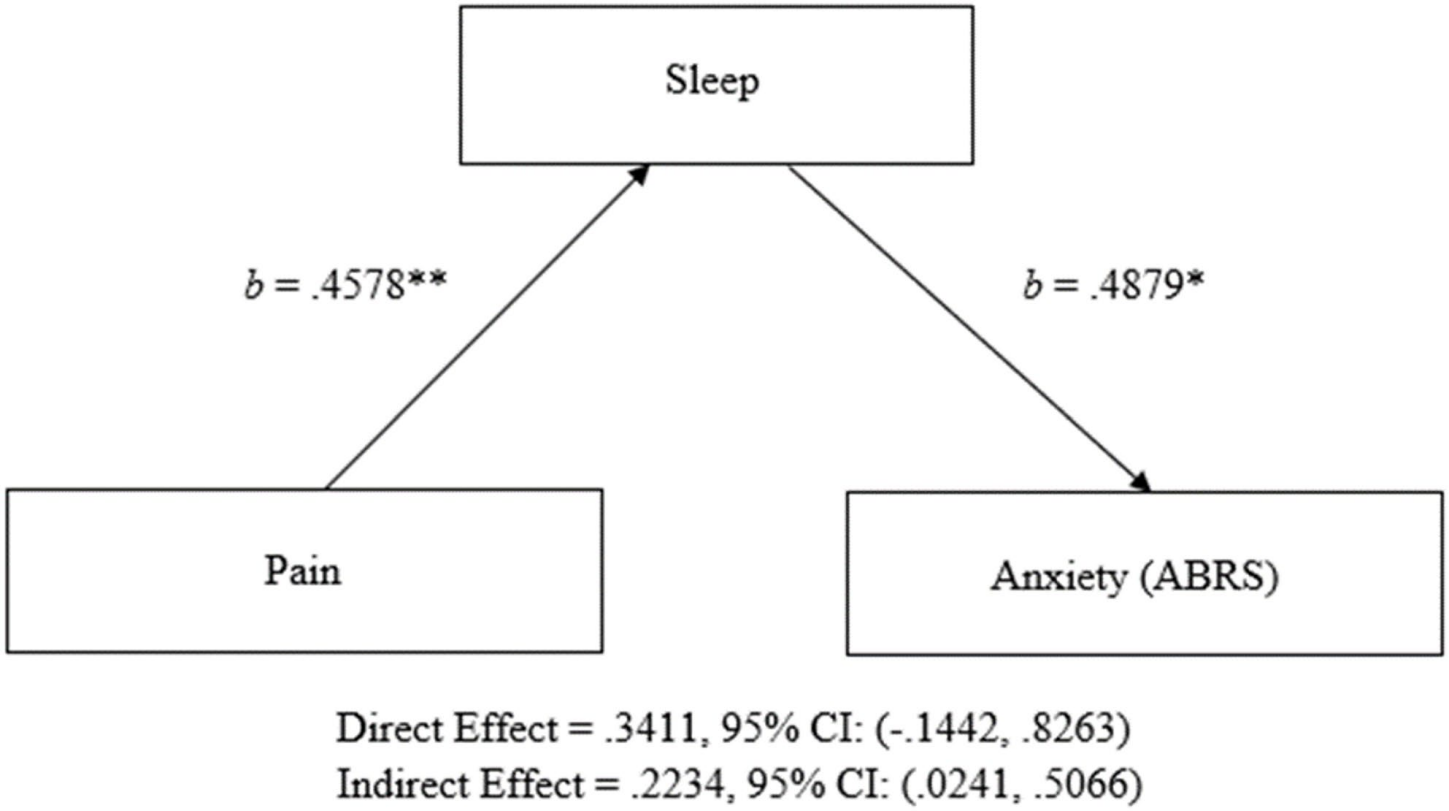
Sleep Mediated Relationship Between Pain and Anxiety in Adults with CHARGE Syndrome. *Note.* The mediation analysis was conducted using PROCESS model 4 [45]. Pain = reported pain difficulties, Sleep = reported sleep difficulties, ABRS = DBC-P Anxiety Behavior Rating Scale. Higher scores indicated higher levels of concern. Both *b* are unstandardized regression coefficients. * = *p* <.05; ** = *p* <.005

A mediation analysis was used to investigate if sleep mediates the relationship between pain and anxiety (as measured by the ABRS). The indirect effect was tested using a percentile bootstrap estimation approach implemented with the PROCESS macro Version 3 [45]. These results indicated the indirect coefficient for sleep was significant, β = 0.2234, *SE* = 0.1229, 95% CI = 0.0241, 0.5066. These results suggest that pain increases anxiety by causing increased difficulties with sleep (see Fig. 1).

## Discussion

### Prevalence and presentation of anxiety and OCD

This research project surveyed adults with CHARGE and legal guardians of adults with CHARGE to further analyze anxiety among the population. The results of this survey provided updated information on anxiety diagnoses and anxious behavior among adults with CHARGE. In addition, new results were found to indicate the relationship between anxiety and potential contributing factors of anxious behavior in CHARGE syndrome (i.e., pain and sleep issues).

Previous literature regarding children with CHARGE found small percentages of diagnosed anxiety disorders despite high levels of obsessive-compulsive behavior [9, 14, 15]. The prevalence of anxiety disorders and OCD among adults with CHARGE in this study was compared to national prevalence data from the National Institutes of Mental Health (NIMH) National Comorbidity Study [46]. This data was collected using the World Health Organization Composite International Diagnostic Interview, a structured diagnostic interview. The prevalence of anxiety disorders among adults with CHARGE in this study (50% reporting at least one anxiety disorder or OCD diagnosis; 39% reporting a GAD diagnosis) was higher than the estimated national average for adults (31.1% and 5.7%, respectively; [47, 48]). The results from the GAD-7 screener also indicated much higher potential levels of GAD (63%) than the general population (5.7%; [47]). Moreover, the prevalence of diagnosed OCD among adults with CHARGE (27%) was much higher than the estimated national average for adults (2.3%; [18]) and 34% of the *ADULT* group reported 8 or more OCD traits (the cut-off on the FOCI screener) suggesting that the prevalence of OCD in CHARGE is higher than the general population.

High levels of diagnosed anxiety disorders overall (and specifically GAD) could indicate that the presence of (generalized) anxious behavior should be added to the behavioral phenotype. The results also present an interesting dichotomy between perceived anxiety of adults with CHARGE syndrome and the presence of anxiety diagnoses. Regardless of clinical anxiety diagnoses, participants report anxiety and anxious behavior as very prevalent among adults with CHARGE syndrome.

In other studies, individuals with CHARGE and their families have reported varied percentages of OCD [7, 8] but the prevalence has always been reported as higher than the national prevalence among the general population. Participants’ answers on scales and checklists (DBC-P and FOCI) in this study indicated that, in particular, obsessive-compulsive behavior is very common among adults with CHARGE syndrome. Across these measures, common compulsions reported were consistent with the literature [9, 14, 15]. The results of this study support the continued inclusion of repetitive and obsessive-compulsive behavior as a core component of the CHARGE behavioral phenotype, as it provides a useful description of the behavior among individuals with CHARGE [11].

The current study also found a high prevalence of MDD and ADHD diagnoses among adults with CHARGE. The percentages of these diagnoses were much higher than national estimates [49, 50] and previous literature on mental health diagnoses in CHARGE syndrome [7, 8]. Legal guardians also reported that their adults with CHARGE syndrome had a high prevalence of ASD, similar to studies of children with CHARGE [10]. It is also possible that these diagnoses are common among individuals with CHARGE due to the high prevalence of obsessive-compulsive behavior as both ASD and ADHD are common differential diagnoses for OCD [51]. The most frequently reported obsession and compulsion on the FOCI by adults with CHARGE syndrome seemed to be related to social concerns (i.e., concern for others safety and needing reassurance from others). This is an interesting finding as ASD has been a common diagnosis among individuals with CHARGE Syndrome [10, 15]; indeed, a third of participants in the *LG* subset of this study have a diagnosis of ASD. Parents of children with CHARGE syndrome have frequently reported that teachers and professionals have identified their child as socially uninterested as a potential contributing factor to their ASD diagnosis. However, this obsession with concern for others and the thematic analysis of participant responses indicate that interacting with others is actually of importance to adults with CHARGE. This could indicate that adults with CHARGE syndrome are socially interested but exhibit repetitive thoughts and actions that may be better indicative of OCD than ASD. Research geared towards analysis of the clinical process behind an anxiety diagnosis and other clinical diagnoses (e.g., MDD, ADHD, ASD, etc.) amongst individuals with CHARGE may provide a better indication of what clinicians consider during their assessment and in their conclusions regarding differential diagnoses.

### The relationship between pain, sleep, and anxiety among adults with CHARGE

Experts on the behavior of individuals with CHARGE syndrome have categorized the basis of the behavior into the self-regulation of three major factors: pain, sensory impairment, and anxiety (also known as the Behavior Triangle, [11]). Pain and sleep concerns were high among adults with CHARGE in this study. This result is comparable to previous research on children with CHARGE [10, 21, 22]. Based on the relationship between pain, sleep, and anxiety found in previous literature [9, 19] and the theorized factors behind anxious behavior in CHARGE syndrome it was expected that pain and sleep would have a significant impact on the anxiety of adults with CHARGE syndrome. The significant correlation between pain and anxiety lends evidence to the theory that pain is an important predictor of the anxious behavior of individuals with CHARGE [11]. Additionally, the results also showed that sleep concerns among adults with CHARGE had a strong correlation to both pain and anxiety. A hierarchical regression analysis indicated that sleep mediates the relationship between pain and anxiety. This suggests that increased pain causes increased difficulties with sleep which then predicts an increase in anxiety.

Both symptoms of pain and sleep difficulties have been regularly studied among individuals with CHARGE syndrome [10, 21, 52]. The impact that these factors have on anxious behavior in the community could potentially be present among those with other genetic syndromes – particularly those that have somatic concerns or medical symptoms that produce pain. As other research has reviewed the differences in anxiety between genetic disorders and other neurodevelopmental disorders [10, 29–32] it might also be worth investigating whether pain is a contributing factor to anxiety among these populations.

There are strategies to combat pain. However, sometimes in the case of medical pain with CHARGE, further medical procedures may be needed to reach the source of the pain (e.g., anesthesia for a dental procedure for a cavity). These additional procedures can lead to more anxiety and pain, particularly when individuals with CHARGE frequently have a surplus of surgeries or other invasive medical procedures in their lifetime [21]. Additionally, pain and chronic pain can lead to anticipation or fear of pain among individuals with CHARGE syndrome. Indeed, preliminary research has shown that the increased number of medical procedures has led to post-traumatic stress disorder symptoms in children with CHARGE [53]. Taken together, this indicates that while pain intervention might be a useful way to lower anxiety among adults with CHARGE, use of additional medical procedures to reduce pain may have the unintended side effect of increasing anxiety and stress.

In contrast to medical intervention of pain, there are many evidence-based behavioral interventions (e.g., sleep hygiene routines) and non-invasive medical techniques (e.g., use of melatonin) to improve sleep and several that have been found effective for individuals with CHARGE syndrome. Kennert [52] found in a study of children with CHARGE that positive bedtime routines with scheduled awakening, melatonin treatment, and the combination of these two strategies were viable treatment options to improve sleep outcomes. Sleep and self-regulation of behavior may also be connected [54] which could provide further evidence for sleep being an important part of the behavior triangle for CHARGE syndrome [11]. Therefore, because sleep mediates the relationship between pain and anxiety, it could be possible that sleep interventions would be a feasible strategy for adults with CHARGE and parents of children with CHARGE to decrease anxiety and related behaviors. Previous research indicates that sleep interventions can be effective when implemented by a parent and among children with CHARGE [52] and perhaps can break the cycle of pain and anxiety experienced by individuals with CHARGE while improving self-regulation and avoiding exacerbation of other potential contributing factors of anxiety.

### Limitations and conclusions

It should be noted that the current and previous studies relied on self-report or parent report of mental health diagnoses. There is a possibility that participants were unaware of their child’s mental health diagnoses, therefore, our current estimates on OCD diagnoses may be an underestimate or overestimate. While the results of this study provide a useful description of anxious behavior among adults with CHARGE, parents and legal guardians may not always understand the distinction between experiencing anxiety and having a diagnosed anxiety disorder. Additionally, while self-report is a useful assessment tool for the diagnosis of anxiety, further behavior observation and direct testing of anxiety would likely lead to a more accurate diagnosis than solely relying on the self-reflection of adults with CHARGE syndrome or their caregivers. As such, future studies should attempt to use alternative and more descriptive methods to gather information on anxiety (e.g., interviews, self-report, direct testing, clinical assessment, analysis of clinical documents, etc.).

Accurate description of anxiety among the participants may also have been hindered by the lingering presence and impact of the COVID-19 pandemic. As participant responses were collected during the beginning of the COVID-19 pandemic (Spring/Summer 2020) it is likely that anxious behavior was affected. Indeed, over half of participants indicated that their anxiety had risen since the beginning of the pandemic. Future studies will need to be done to see if the increased levels of anxiety persist or are merely a result of the zeitgeist. Regardless, researching and developing effective anxiety interventions for individuals with CHARGE is important and will continue to be important (perhaps even more so if the pandemic has led to a permanent increase in anxious behavior).

The major findings of this research focused on the *frequency* of pain and sleep concerns in relation to anxiety symptoms. While we gathered specific behaviors related to anxiety and obsessive-compulsive disorder in this survey, we did not gather more specific data as to the specific *type* of issues for either sleep nor pain as previous research in CHARGE syndrome has [21, 52]. This limited the specificity to which we could correlate specific pain and sleep behaviors to anxiety behaviors. Future research might consider this further and results would provide greater insight into which particular pain or sleep factors would be good candidates for intervention in order to reduce anxiety symptoms.

Sensory impairment and communication difficulties provide particular challenges to the current research. It may be difficult for adults with CHARGE and their caregivers (and professionals for that matter) to differentiate between what is true anxiety, rational fear, and which behaviors are filling sensory or communicative needs. For example, children with CHARGE syndrome may engage in repetitive checking behaviors more frequently as a functional way to compensate for their compromised vision and auditory systems and to navigate their environment [11]. If it is difficult for those with an expertise in CHARGE syndrome to make these distinctions, it is reasonable to assume that other legal guardians and professionals with limited exposure to CHARGE, genetic syndromes, and sensory impairment may have difficulties in truly understanding anxiety within CHARGE syndrome. Research geared towards analysis of the clinical process behind an anxiety diagnosis and other clinical diagnoses (e.g., MDD, ADHD, ASD, etc.) may provide a better indication of what clinicians consider during their assessment and in their conclusions regarding clinical diagnoses and the interventions that they recommend. Further exploration of alternative diagnostic models (e.g., RDoC) could offer a more defined or tailored approach to diagnosing anxiety among individuals with CHARGE. Additionally, researched causes of OCD in the general population (e.g., cortico-striato-thalamo-cortical loop, serotonin or dopamine levels, maladaptive learning based on obsessive anxiety, etc.; [55]) have not been studied directly in CHARGE syndrome yet. While the current study provided some indication that pain and sleep are implicated in the anxious behavior among the population, other potential causes of OCD in CHARGE were not able to be thoroughly analyzed (e.g., sensory impairment). Future studies focused on the specific etiology related to OCD in CHARGE syndrome would be helpful for differential diagnosis and treatment of this disorder.

Research suggests that high rates of anxiety are prevalent among other developmental disorders and genetic syndromes [29, 30, 32]. In addition, there is growing evidence to suggest that different genetic syndromes exhibit differential presentations of specific anxiety disorders [29]. Although not yet empirically supported, it is possible that CHARGE syndrome might also have uniquely higher levels of a particular anxiety disorder (namely OCD) or higher levels of behavior characteristic of particular subtypes of anxiety. Indeed, preliminary results from previous research [10] indicated that children and adolescents with CHARGE syndrome received higher OCD subscale scores than individuals with other genetic syndromes and typically developing children with an anxiety diagnosis. There were no significant differences between the anxiety scores between children with CHARGE [10] and adults with CHARGE (current study) indicating that the increased levels of anxiety and behaviors characteristic of OCD are prevalent across the community regardless of age. Further research is necessary to measure, describe, and compare the anxiety of adults with CHARGE to children and adolescents with CHARGE syndrome and to individuals with other genetic syndromes.

Anxiety is a significant concern within the CHARGE community. The current study was designed to gain a thorough description of the anxiety diagnoses and behaviors of adults with CHARGE syndrome and how it can be influenced by potential contributing factors of anxiety. Professionals working with clients with CHARGE should consider the multitude of unique factors influencing the expression of anxiety symptoms among these individuals, particularly pain and sleep. Future research should continue to study the clinical process in anxiety diagnosis among the CHARGE community and methods to increase access to anxiety interventions that are effective and adapted to the needs of individuals with CHARGE syndrome – such as sleep interventions.

## Data Availability

The data sets generated during the current study are not publicly available because the participants of this study did not give written consent for their data to be shared publicly and our Institutional Review Board advised the researchers that the data could contain information that could compromise the privacy of research participants.

## Abbreviations

ADHD: Attention- deficit/hyperactivity disorder
ASD: Autism spectrum disorder
ABRS: Anxiety Behavior Rating Scale from the DBC-P
ADULT group: Participants who were adults with CHARGE Syndrome
CHARGE: CHARGE Syndrome
CHD7: Chromodomain helicase DNA binding protein 7
COMBINED group: All participants, which included both the Adult and LG participants
DBC-P: Developmental Behavior Checklist-Parent Version
DSM-5: Diagnostic and Statistical Manual of Mental Disorders, 5th Edition
FOCI: Florida Obsessive-Compulsive Inventory
GAD: Generalized anxiety disorder
GAD-7: Generalized Anxiety Disorder 7-Item
LG group: Participants who were legal guardians of an adult with CHARGE Syndrome
MDD: Major depressive disorder
OCD: Obsessive-compulsive disorder

## References

[1] Janssen N, Bergman JEH, Swertz MA, Tranebjaerg L, Lodahl M, Schoots J, Hofstra RMW, Ravenswaaij-Arts CMA, Hoefsloot LH. Mutation update on the CHD7 gene involved in CHARGE syndrome. Hum Mutat. 2012;33(8):1149–60. 10.1002/humu.22086. 22461308 10.1002/humu.22086

[2] Blake KD, Davenport SLH, Hall BD, Hefner MA, Pagon RA, Williams MS, et al. Charge association: an update and review for the primary pediatrician. Clin Pediatr (Phila). 1998;37(3):159–73. 10.1177/000992289803700302. 9545604 10.1177/000992289803700302

[3] Pagon RA, Graham JM, Zonana J, Yong SL. Coloboma, congenital heart disease, and choanal atresia with multiple anomalies: CHARGE association. J Pediatr. 1981;99(2):223–7. 10.1016/s0022-3476(81)80454-4. 6166737 10.1016/s0022-3476(81)80454-4

[4] Hefner MA. Introduction to CHARGE syndrome. In: Hartshorne TS, Hefner MA, Blake KD, editors. CHARGE syndrome. 2nd ed. San Diego (CA): Plural Publishing; 2021. pp. xi–xvi.

[5] Salem-Hartshorne N. Cognitive development. In: Hartshorne TS, Hefner MA, Blake KD, editors. CHARGE syndrome. 2nd ed. San Diego (CA): Plural Publishing; 2021. pp. 253–9.

[6] Wiley S. Psychiatric treatment. In: Hartshorne TS, Hefner MA, Blake KD, editors. CHARGE syndrome. 2nd ed. San Diego (CA): Plural Publishing; 2021. pp. 425–38.

[7] Blake KD, Salem-Hartshorne N, Daoud MA, Gradstein J. Adolescent and adult issues in CHARGE syndrome. Clin Pediatr (Phila). 2005;44(2):151–9. 10.1177/000992280504400207. 15735833 10.1177/000992280504400207

[8] Wachtel LE, Hartshorne TS, Dailor AN. Psychiatric diagnoses and psychotropic medications in CHARGE syndrome: a pediatric survey. J Dev Phys Disabil. 2007;19(5):471–83. 10.1007/s10882-007-9064-6.

[9] Hartshorne N, Hudson A, MacCuspie J, Kennert B, Nacarato T, Hartshorne T, et al. Quality of life in adolescents and adults with CHARGE syndrome. Am J Med Genet A. 2016;170(8):2012–21. 10.1002/ajmg.a.37769. 27273681 10.1002/ajmg.a.37769

[10] Madhavan-Brown SA. Anxiety of individuals with CHARGE syndrome [master’s thesis]. Mount Pleasant (MI): Central Michigan University; 2019.

[11] Hartshorne TS, Stratton KK, Brown D, Madhavan-Brown SA, Schmittel MC. Behavior in CHARGE syndrome. Am J Med Genet A. 2017;175(4):431–8. 10.1002/ajmg.c.31588. 10.1002/ajmg.c.31588 29082623

[12] Anxiety and Depression Association of America. Understanding disorders: What are anxiety and depression? [Internet]. Maryland: ADAA [cited 2022 Apr]. Available from: https://adaa.org/understanding-anxiety

[13] National Institute of Mental Health. Anxiety Disorders [Internet]. Maryland: NIMH [cited 2024 Dec]. Available from: https://www.nimh.nih.gov/health/topics/anxiety-disorders/index.shtml

[14] Bernstein V, Denno LS. Repetitive behaviors in CHARGE syndrome: differential diagnosis and treatment options. Am J Med Genet A. 2005;133(3):232–9. 10.1002/ajmg.a.30542. 10.1002/ajmg.a.30542 15688425

[15] Hartshorne TS, Cypher AD. Challenging behavior in CHARGE syndrome. Ment Health Aspects Dev Disabil. 2004;7(2):41–52.

[16] Gedye A. Recognizing obsessive-compulsive disorder in clients with developmental disabilities. Habilit Ment Healthc Newsl. 1992;11(1):73–7.

[17] Hartshorne TS. Behavior. In: Hartshorne TS, Hefner MA, Blake KD, editors. CHARGE syndrome. 2nd ed. San Diego (CA): Plural Publishing; 2021. pp. 413–24.

[18] National Institute of Mental Health. Statistics: Obsessive-compulsive disorder (OCD) [Internet]. Maryland; NIMH. [cited 2022 Apr]. Available from: https://www.nimh.nih.gov/health/statistics/obsessive-compulsive-disorder-ocd

[19] May ME, Kennedy CH. Health and problem behavior among people with intellectual disabilities. Behav Anal Pract. 2010;3(2):4–12. 10.1007/BF03391759. 22532888 10.1007/BF03391759 PMC3004690

[20] Stratton KK. Pain. In: Hartshorne TS, Hefner MA, Blake KD, editors. CHARGE syndrome. 2nd ed. San Diego (CA): Plural Publishing; 2021. pp. 439–48.

[21] Stratton KK, Hartshorne T. Identifying pain in children with CHARGE syndrome. Scand J Pain. 2019;19(1):157–66. 10.1515/sjpain-2018-0080. 30226210 10.1515/sjpain-2018-0080

[22] Hartshorne TS, Heussler HS, Dailor AN, Williams GL, Papadopoulos D, Brandt KK. Sleep disturbances in CHARGE syndrome: types and relationships with behavior and caregiver well-being. Dev Med Child Neurol. 2009;51(2):143–50. 10.1111/j.1469-8749.2008.03146.x. 19018833 10.1111/j.1469-8749.2008.03146.x

[23] Gregory AM, Eley TC. Sleep problems, anxiety and cognitive style in school-aged children. Infant Child Dev. 2005;14(5):435–44. 10.1002/icd.409.

[24] Hefner MA, Davenport SLH. Overview and sensory issues. In: Hartshorne TS, Hefner MA, Blake KD, editors. CHARGE syndrome. 2nd ed. San Diego (CA): Plural Publishing; 2021. pp. 3–13.

[25] Swanson LA. Communication: the speech and Language perspective. In: Hartshorne TS, Hefner MA, Blake KD, editors. CHARGE syndrome. 2nd ed. San Diego (CA): Plural Publishing; 2021. pp. 329–52.

[26] Pinquart M, Shen Y. Anxiety in children and adolescents with chronic physical illnesses: a meta-analysis. Acta Paediatr. 2011;100(8):1069–76. 10.1111/j.1651-2227.2011.02223.x. 21332786 10.1111/j.1651-2227.2011.02223.x

[27] Hartshorne TS, Schmittel MC. Social-emotional development in children and youth who are deafblind. Am Ann Deaf. 2016;161(4):444–53. 10.1353/aad.2016.0036. 27818400 10.1353/aad.2016.0036

[28] Stratton KK. Stress. In: Hartshorne TS, Hefner MA, Blake KD, editors. CHARGE syndrome. 2nd ed. San Diego (CA): Plural Publishing; 2021. pp. 449–54.

[29] Crawford H, Waite J, Oliver C. Diverse profiles of anxiety related disorders in fragile X, Cornelia de Lange, and Rubinstein-Taybi syndromes. J Autism Dev Disord. 2017;47(12):3728–40. 10.1007/s10803-016-3015-y. 28144878 10.1007/s10803-016-3015-y PMC5676833

[30] Dankner N, Dykens EM. Anxiety in intellectual disabilities: challenges and next steps. Int Rev Res Dev Disabil. 2012;42:57–83. 10.1016/b978-0-12-394284-5.00003-6.

[31] Groves L, Moss J, Oliver C, Royston R, Waite J, Crawford H. Divergent presentation of anxiety in high-risk groups within the intellectual disability population. J Neurodev Disord. 2022. 10.1186/s11689-022-09462-w. 36199025 10.1186/s11689-022-09462-w PMC9535841

[32] Leyfer O, Woodruff-Borden J, Mervis CB. Anxiety disorders in children with Williams syndrome, their mothers, and their siblings: implications for the etiology of anxiety disorders. J Neurodev Disord. 2009;1(1):4–14. 10.18297/etd/821. 20161441 10.1007/s11689-009-9003-1 PMC2790165

[33] Einfeld SL, Tonge BJ. Manual for the developmental behaviour checklist: primary carer version (DBC-P) and teacher version (DBC-T). 2nd ed. Monash University Centre for Developmental Psychiatry and Psychology; 2002.

[34] Spitzer RL, Kroenke K, Williams JB, Lowe B. A brief measure for assessing generalized anxiety disorder: the GAD-7. Arch Intern Med. 2006;166(10):1092–7. 10.1001/archinte.166.10.1092. 16717171 10.1001/archinte.166.10.1092

[35] Salem-Hartshorne N, Jacob S. Characteristics and development of children with CHARGE association/syndrome. J Early Interv. 2004;26(4):292–301. 10.1177/105381510402600405.

[36] Verloes A. Updated diagnostic criteria for CHARGE syndrome: a proposal. Am J Med Genet A. 2005;133(3):306–8. 10.1002/ajmg.a.30559. 10.1002/ajmg.a.30559 15666308

[37] Storch EA, Kaufman DA, Bagner D, Merlo LJ, Shapira NA, Geffken GR, et al. Florida obsessive-compulsive inventory: development, reliability, and validity. J Clin Psychol. 2007;63(9):851–9. 10.1002/jclp.20382. 17674398 10.1002/jclp.20382

[38] Rosenfeld R, Dar R, Anderson D, Kobak KA, Greist JH. A computer-administered version of the Yale-Brown Obsessive-Compulsive scale. Psychol Assess. 1992;4(3):329–32.

[39] Aleda MA, Geffken GR, Jacob ML, Goodman WK, Storch EA. Further psychometric analysis of the Florida obsessive-compulsive inventory. J Anxiety Disord. 2009;23(1):124–9. 10.1016/j.janxdis.2008.05.001. 18571371 10.1016/j.janxdis.2008.05.001

[40] Goodman WK, Price LH, Rasmussen SA, Mazure C, Fleischmann RL, Hill CL, et al. The Yale-Brown obsessive compulsive scale: I. development, use, and reliability. Arch Gen Psychiatry. 1989;46(11):1006–11. 10.1001/archpsyc.1989.01810110048007. 2684084 10.1001/archpsyc.1989.01810110048007

[41] Plummer F, Manea L, Trepel D, McMillan D. Screening for anxiety disorders with the GAD-7 and GAD-2: a systematic review and diagnostic meta-analysis. Gen Hosp Psychiatry. 2016;39:24–31. 10.1016/j.genhosppsych.2015.11.005. 26719105 10.1016/j.genhosppsych.2015.11.005

[42] Beck AT, Epstein N, Brown G, Steer RA. An inventory for measuring clinical anxiety: psychometric properties. J Consult Clin Psychol. 1988;56(6):893–7. 10.1037/0022-006x.56.6.893. 3204199 10.1037//0022-006x.56.6.893

[43] Derogatis LR, Lipman RS, Rickels K, Uhlenhuth EH, Covi L. The Hopkins symptom checklist (HSCL): a self-report symptom inventory. Behav Sci. 1974;19(1):1–15. 10.1002/bs.3830190102. 4808738 10.1002/bs.3830190102

[44] Spitzer RL, Williams JB, Kroenke K, Hornyak R, McMurray J. Validity and utility of the PRIME-MD patient health questionnaire in assessment of 3000 obstetric-gynecologic patients: the PRIME-MD patient health questionnaire obstetrics-gynecology study. Am J Obstet Gynecol. 2000;183(3):759–69. 10.1067/mob.2000.106580. 10992206 10.1067/mob.2000.106580

[45] Hayes AF. Introduction to mediation, moderation, and conditional process analysis: A regression-based approach. New York: The Guilford Press; 2018.

[46] Kessler RC, Üstün TB, The World Mental Health (WMH) Survey Initiative version of the World Health Organization (WHO) Composite International Diagnostic Interview (CIDI). International Journal of Methods in Psychiatric Research [Internet]. 2021;13(2):93–121. Available from: https://onlinelibrary.wiley.com/doi/full/10.1002/mpr.168 10.1002/mpr.168 PMC6878592 15297906

[47] National Institute of Mental Health. Statistics: Generalized anxiety disorder (GAD) [Internet]. Maryland; NIMH. [cited 2022 Apr]. Available from: https://www.nimh.nih.gov/health/statistics/generalized-anxiety-disorder

[48] National Institute of Mental Health. Statistics: Any anxiety disorders [Internet]. Maryland; NIMH. [cited 2022 Apr]. Available from: https://www.nimh.nih.gov/health/statistics/any-anxiety-disorder.shtml

[49] Kessler RC, Adler L, Barkley R, Biederman J, Conners CK, Demler O, et al. The prevalence and correlates of adult ADHD in the United States: results from the National Comorbidity Survey Replication. Am J Psychiatry. 2006;163(4):716–23. 10.1176/ajp.2006.163.4.716. 16585449 10.1176/appi.ajp.163.4.716 PMC2859678

[50] National Institute of Mental Health. Major Depression [Internet]. Maryland; NIMH. [cited 2022 Apr]. Available from: https://www.nimh.nih.gov/health/statistics/major-depression

[51] Krebs G, Heyman I. Obsessive-compulsive disorder in children and adolescents. Arch Dis Child. 2015;100(5):495–9. 10.1136/archdischild-2014-306934. 25398447 10.1136/archdischild-2014-306934 PMC4413836

[52] Kennert BA. Investigation of two methods for treating sleep problems among children with CHARGE syndrome [dissertation]. Mount Pleasant (MI): Central Michigan University; 2018.

[53] Sykes SM. Medical experiences and subsequent behaviors in children and adolescents with CHARGE syndrome. [master’s thesis]. Mount Pleasant (MI): Central Michigan University; 2021.

[54] Kroese FM, De Ridder DT, Evers C, Adriaanse MA. Bedtime procrastination: introducing a new area of procrastination. Front Psychol. 2014;5:611. 10.3389/fpsyg.2014.00611. 24994989 10.3389/fpsyg.2014.00611 PMC4062817

[55] Brock H, Rizvi A, Hany M. Obsessive-Compulsive Disorder (OCD). [Internet]. PubMed. Treasure Island (FL): StatPearls Publishing; 2024. Available from: https://www.ncbi.nlm.nih.gov/books/NBK553162/31985955

